# Secretory Breast Carcinoma: A Rare Breast Cancer With an Excellent Behavior

**DOI:** 10.7759/cureus.73312

**Published:** 2024-11-09

**Authors:** Karich Nassira, Anass Haloui, Chaimae Bekhakh, Noura Seghrouchni, Amal Bennani

**Affiliations:** 1 Pathology, Mohammed VI University International Hospital, Oujda, MAR; 2 Pathology, Faculty of Medicine, Mohammed VI University International Hospital, Mohamed I University, Oujda, MAR; 3 Pathology, Mohammed VI University International Hospital, Mohamed I University, Oujda, MAR

**Keywords:** adjuvant radiotherapy, breast cancer, case report, chemotherapy, secretory breast carcinoma

## Abstract

Secretory breast carcinoma (SBC) is a rare breast cancer subtype, histologically, defined by abundant eosinophilic secretions with a triple-negative staining on immunohistochemistry. The diagnosis is made histologically and often requires complementary methods such as immunohistochemical studies and fluorescence in situ hybridization (FISH). Surgery, whether conservative or radical, is the preferred treatment. The prognosis remains good. We report a case of a 46-year-old woman who presented with a right breast upper quadrant lump. She underwent a mastectomy with axillary curage. The pathological result was in favor of a breast secretory carcinoma. The lack of any poor prognostic factors allowed the patient to be placed on close follow-up with a good outcome and no recurrence or metastasis in the last two years.

## Introduction

Secretory breast carcinoma (SBC), previously known as juvenile breast carcinoma, is a rare malignancy, representing less than 0.15% of all breast cancers [1]. The first description was in 1966 by McDivitt and Stewart [2]. SBC is characterized by a t (12;15) translocation, causing fusion of the ETV6 and NTRK3 genes, with consequent abnormal cell proliferation [3]. This breast cancer type is characterized by an indolent clinical course. Histologically, it is distinguished by abundant eosinophilic secretions with triple-negative staining on immunohistochemistry [4]. We report the case of a 46-year-old woman diagnosed with secretory carcinoma of the breast with a favorable course.

## Case presentation

**Figure 1:**
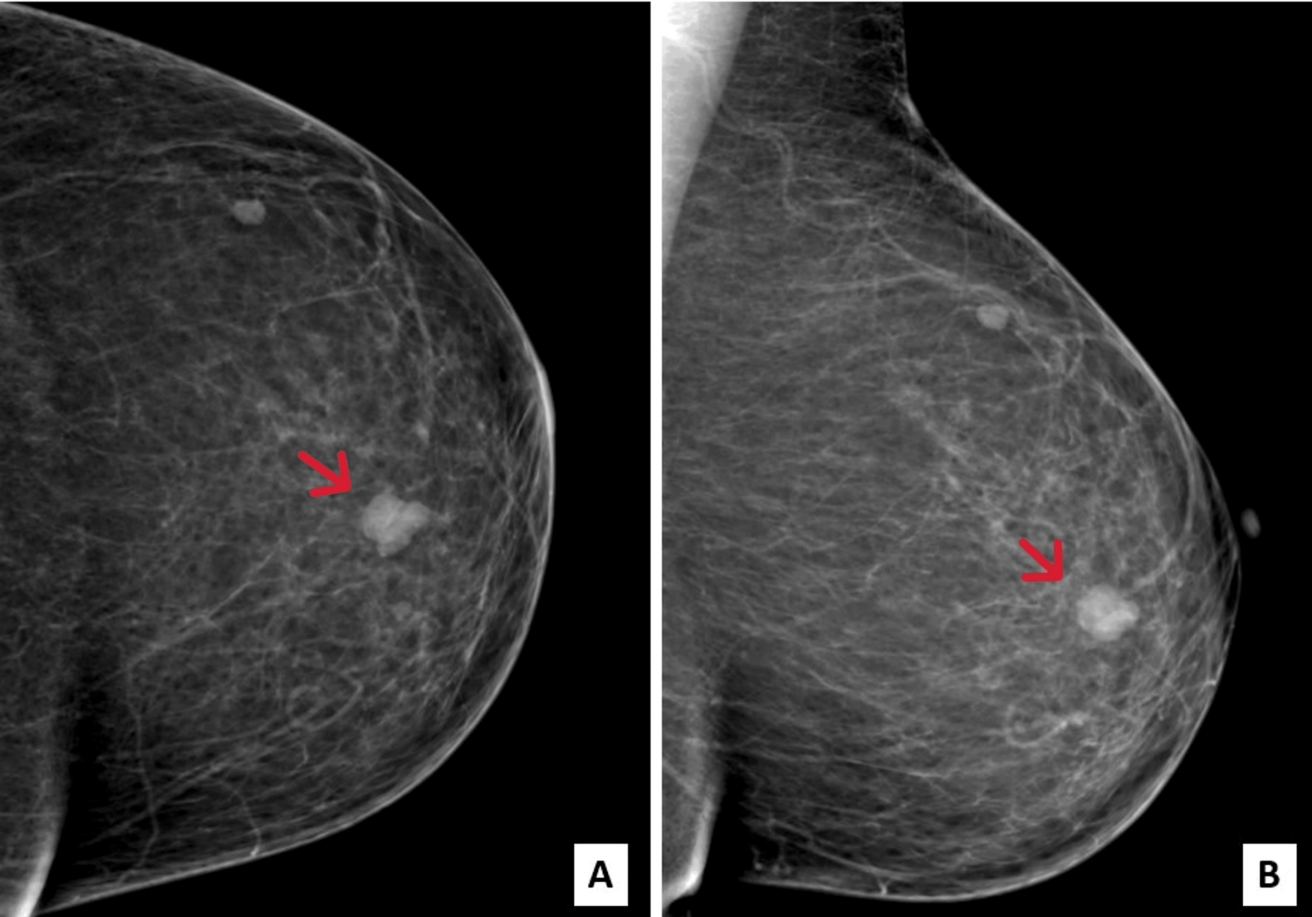
Mammography showed a well-limited, polylobed opacity in the lower inner quadrant of the right breast, measuring 15x12 mm (red arrows). (A) Right craniocaudal incidence. (B) Right mediolateral oblique incidence.

**Figure 2:**
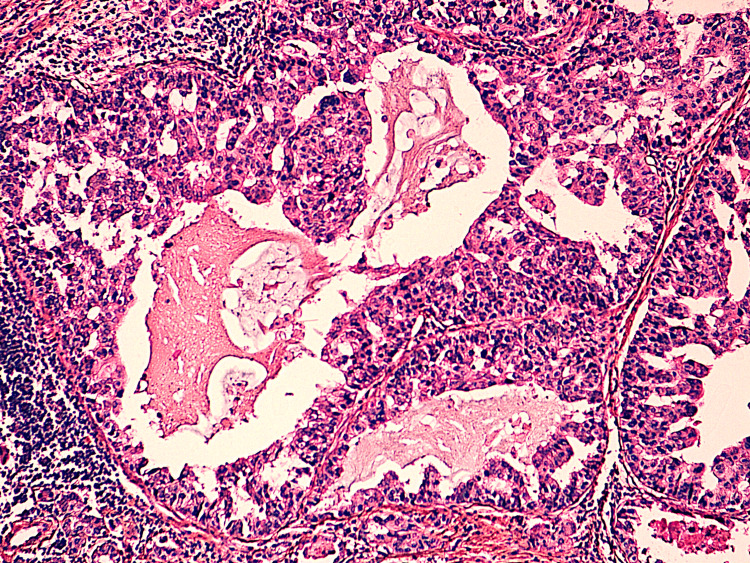
Micrograph showing tumor proliferation with dilated microcystic glands; tumor cells show moderate atypia (H&E x40). H&E: hematoxylin and eosin.

We report a case of a 46-year-old woman with no pathological history who presented with a right breast upper quadrant lump. The lesion was firm and painless, with limited mobility. There was no nipple discharge, spontaneous or on pressure. In addition, the patient reported a slight increase in size throughout the two months of evolution. The patient did not report weight loss or pain. Clinical examination did not find axillary lymph nodes. Mammography revealed two breast lesions: the first was a well-limited, polylobed opacity in the lower inner quadrant, measuring 15x12 mm, classified as Breast Imaging Reporting and Data System (BIRADS) 4; the second was a small, well-limited opacity in the upper outer quadrant, measuring 7x4 mm, classified as BIRADS 3 (Figure 1). Ultrasound performed in complement to mammography revealed a polylobed, hypoechoic lesion with posterior attenuation. Subsequently, the Tru-cut biopsy of the first lesion showed a well-differentiated breast carcinoma made up of well-differentiated glandular structures and trabeculae showing moderate atypia and mitoses (Figure 2). Initially, it was interpreted as an SBR grade 2 infiltrating ductal carcinoma. The tumor was estrogen receptor (ER) and progesterone re­ceptor (PR) negative and HER2-negative. We also performed a microbiopsy of the second lesion, which was consistent with an adenofibroma.

Thoraco-abdomen-pelvic CT scan shows no evidence of metastatic lesions. Surgery was decided upon, and the patient underwent a right mastectomy with homolateral axillary lymph node dissection.

**Figure 3:**
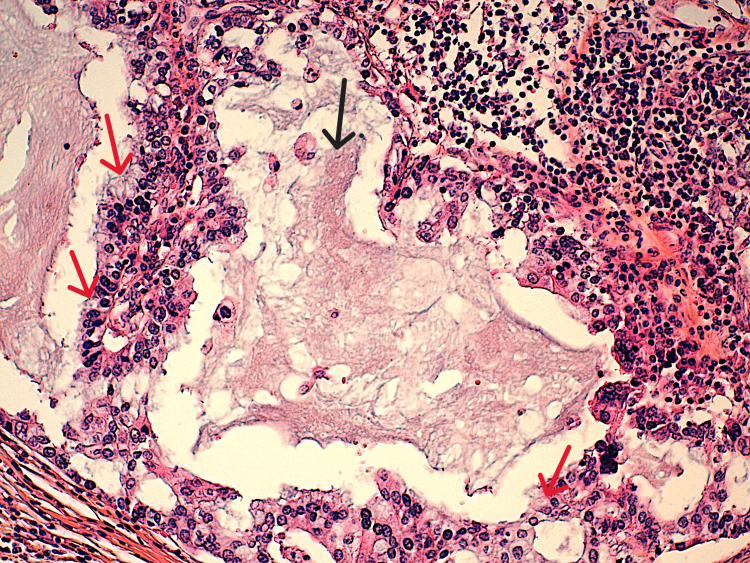
Histological image showing dilated glands filled with eosinophilic secretions (black arrow); these secretions are also present in the intracellular spaces (red arrows) (H&E x40). H&E: hematoxylin and eosin.

**Figure 4:**
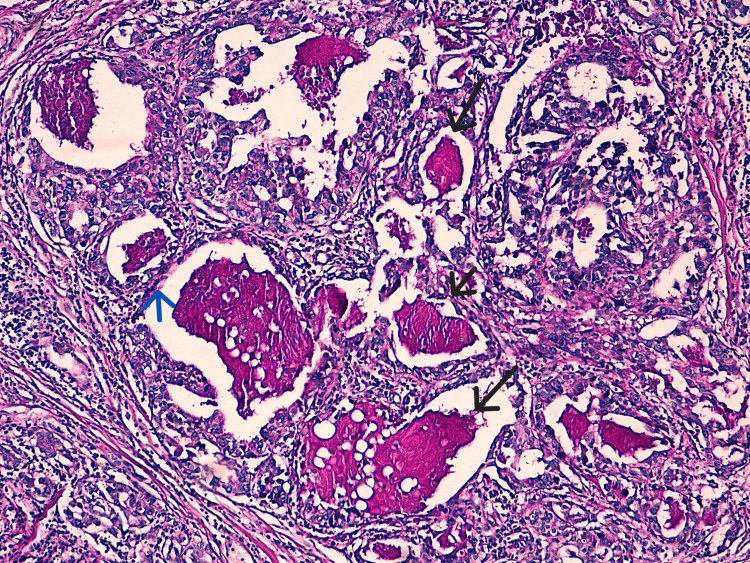
Histological image after PAS staining showing PAS-positive secretions intracellular (blue arrow) and in the glandular lumens (black arrows) (PAS x40). PAS: periodic acid-Schiff.

Histological examination of the specimen showed a circumscribed carcinomatous proliferation, consisting essentially of glands and trabeculae. Tumor cells showed moderate atypia and low mitotic activity. The special feature of this tumor is the presence of tumor cells with vacuolated, foamy cytoplasm and abundant intracellular pale blue to dense pink secretions (Figure 3), which are periodic acid-Schiff (PAS) positive and diastase resistant (Figure 4). An in-situ component was noted. No vascular emboli or peri-nervous sheathing was observed. All lymph nodes were free of tumor involvement. The nipple and skin were not infiltrated, and there was no Paget's disease. The immunohistochemical study was repeated, confirming the triple-negative subtype. Therefore, the diagnosis of secretory carcinoma was made on histological criteria. Translocation research was not carried out due to lack of financial resources.

The patient was subsequently placed under close medical surveillance with good elaboration. Currently, she has not developed any local recurrence or metastasis after two years of follow-up.

## Discussion

Secretory breast carcinoma (SBC), previously known as juvenile breast carcinoma, is a rare malignancy, representing less than 0.15% of all breast cancers [1]. The first description was in 1966 by McDivitt and Stewart [2]. The term juvenile breast carcinoma was later changed by Tavassoli and Norris in 1980, who reviewed 19 cases with ages ranging from nine to 69 years [4]. Subsequently, many case reports were published confirming this fact, with an average age of 25 (3-83) [5,6]. A distinct female predominance is noted, with only rare cases reported in men (18 cases) [7].

Clinically, the studies report a firm, painless mass, often located in the upper outer quadrants. Occasionally, with nipple discharge [5]. In our case, however, the mass was in the lower inner quadrant. This tumor is most often detected as a small, benign-looking nodule or group of nodules or as an intraductal lesion [8].

SBC usually has the appearance of a well-circumscribed mass with a firm consistency and a grayish-white to tan-brown color. Its size is variable, reaching up to 16 cm [9].

The pathological features of this tumor are characteristic and are characterized by an abundant eosinophilic secretion in intracellular vacuoles and in­tercellular spaces. These secretions are periodic acid-Schiff (PAS) positive. SBC has three main architectures: tubular, solid, and microcystic, or a mixture of the three. Tumor cells show mild cytologic atypia with uniform nuclei and low mitotic activity [10]. On immunohistochemistry, the tumor has a triple-negative profile, but low positivity of hormone receptors is sometimes found. However, some studies have shown moderate to high expression of hormone receptors. The tumor also shows reactivity with the S-100 protein [11,12]. The present case was in line with the literature, showing a triple-negative profile.

The differential diagnosis is mainly with invasive breast carcinoma of no special type (NOS), formerly known as invasive ductal carcinoma. Indeed, in the two tumor types, we find tubular structures and desmoplastic tumor stroma with blood vessel elastosis. The detection of PAS-positive secretions in intracellular vacuoles and in­tercellular spaces is a crucial element in the diagnosis. However, invasive ductal carcinoma can sometimes show mucin secretion, which makes the diagnosis more difficult. In these cases, molecular biology techniques are essential [10]. The SBC is characterized by pathognomonic ETV6-NTRK3 gene fusion, which can be confirmed by RT-PCR using ETV6 and NTRK3 primers, or by fluorescence in situ hybridization (FISH) using either ETV6 break-apart probes or 5’ ETV6 and 3’ NTRK3 convergence probes. Also, the development of molecular biology techniques has helped identify the pan-TRK protein by immunohistochemistry, which is becoming a promising lead in diagnosis [13].

The management of SBC takes into account several factors, including patient age, hormone positivity on immunohistochemistry, lymph node involvement, and tumor size [14]. Surgery, whether conservative or radical, is the preferred treatment for this type of tumor [15,16]. The efficacy of chemotherapy remains debatable, given the limited number of studies on this type of cancer [17]. The same for adjuvant radiotherapy, whose efficacy remains limited and is mainly indicated for conservative treatment [15,17,18]. On the other hand, hormone therapy can be useful, especially in patients where estrogen or progesterone receptors are expressed [17]. Our patient was treated radically, with no lymph node metastases or distant metastases and no expression of hormone receptors. Furthermore, the size of the tumor was small, so doctors opted for long-term follow-up.

The overall tumor prognosis is excellent, with a five-year survival rate exceeding 95% [17]. However, the prognosis becomes poorer when the tumor presents at an inflammatory stage before initial treatment [19,20].

## Conclusions

SBC is a rare breast cancer subtype with an excellent prognosis. Histologically, it is distinguished by abundant eosinophilic secretions with a triple-negative staining on immunohistochemistry. The diagnosis is made histologically and often requires complementary methods such as immunohistochemical studies and FISH. Surgery, whether conservative or radical, is the preferred treatment. The benefits of chemotherapy and radiotherapy remain debatable. Management is not codified, given the rarity of this subtype, so further studies are needed in order to establish appropriate management.
